# An Internet of Vehicles (IoV) Access Gateway Design Considering the Efficiency of the In-Vehicle Ethernet Backbone

**DOI:** 10.3390/s21010098

**Published:** 2020-12-25

**Authors:** Dae-Young Kim, Minwoo Jung, Seokhoon Kim

**Affiliations:** 1 School of Computer Software, Daegu Catholic University, Gyeongsan 38430, Korea; kimdy81@cu.ac.kr; 2 R&D Center LiDAR Development Team, Carnavicom Co., Ltd., Incheon 21984, Korea; jungminwoo80@gmail.com; 3 Department of Software Convergence, Soonchunhyang University, Asan 31538, Korea

**Keywords:** in-vehicle network, vehicle gateway, Ethernet backbone, traffic control, Internet of Vehicles

## Abstract

A vehicular network is composed of an in-vehicle network (IVN) and Internet of Vehicles (IoV). IVN exchanges information among in-vehicle devices. IoV constructs Vehicle-to-X (V2X) networks outside vehicles and exchanges information among V2X elements. These days, in-vehicle devices that require high bandwidth is increased for autonomous driving services. Thus, the spread of data for vehicles is exploding. This kind of data is exchanged through IoV. Even if the Ethernet backbone of IVN carries a lot of data in the vehicle, the explosive increase in data from outside the vehicle can affect the backbone. That is, the transmission efficiency of the IVN backbone will be reduced due to excessive data traffic. In addition, when IVN data traffic is transmitted to IoV without considering IoV network conditions, the transmission efficiency of IoV is also reduced. Therefore, in this paper, we propose an IoV access gateway to controls the incoming data traffic to the IVN backbone and the outgoing data traffic to the IoV in the network environment where IVN and IoV are integrated. Computer simulations are used to evaluate the performance of the proposed system, and the proposed system shows better performance in the accumulated average transmission delay.

## 1. Introduction

Automotive technologies are advancing for autonomous driving. To realize autonomous driving, a lot of vehicle-related data is required. This leads to the need for more sensors in a vehicle. More electric control units (ECUs) are needed to control the vehicle through the sensor data. The increased sensors in vehicles generate a lot of data and the increased ECUs consume the data from sensors. In such a change in the environment of vehicle technology, in-vehicle networks (IVNs) are becoming important due to an increase in the amount of data consumed in the vehicle. In-vehicle device modules (e.g., sensors and ECUs) construct domains according to their functions, and data generated in each domain is exchanged between domains. In particular, massive data is generated by cameras and light detection and ranging (LiDAR) sensors for autonomous driving and by multimedia for infotainment services. Therefore, a high-speed IVN backbone is needed for this kind of data transmission [1,2,3]. 

As IVN communication technology, various technologies have been applied according to the type of data traffic in the vehicle. These include the local interconnect network (LIN), controller area network (CAN), CAN-flexible data rate (CAN-FD), media-oriented system transport (MOST), FlexRay, and Ethernet. LIN provides very low-speed data (20 Kbps) communication. LIN is composed of a master and slaves and is used for communication to control elements of the vehicle body such as the glass, doors, wipers, etc. CAN provides conventional in-vehicle communication between sensors and ECUs for powertrain. CAN provides low bandwidth (1 Mbps) for communication. Because of this bandwidth limitation, CAN-FD is proposed. CAN-FD expands the size of transmitted data and improves the bandwidth. FlexRay is a bus system with high-speed (10 Mbps) communication. FlexRay is used for the safety controls in a vehicle. Both MOST and Ethernet are communication technologies that transmit multimedia or infotainment traffic in vehicles over a high bandwidth [4,5,6,7,8,9,10,11,12]. Recently, the use of Ethernet is expanding to handle large amounts of autonomous driving traffic at a high speed. Ethernet backbone is required for high-speed processing of data generated in each functional domain of a vehicle. 

The IEEE defines IEEE 802.3 bw (100BASE-T1) [13] and IEEE 802.3 bp (1000BASE-T1) [14] as Ethernet communication for the IVN backbone. 100BASE-T1 (100Mbps) and 1000BASE-T1 (1 Gbps) support high-speed communication in the backbone, and the application of 1000BASE-T1 is expanding. The Ethernet communication uses CSMA/CD for media access control. Although various vehicle traffic can be reliably transmitted through the vehicle Ethernet, a way to guarantee transmission of time-sensitive data is still required. Thus, the IEEE 802 group proposed a time-sensitive networking (TSN) standard that improves conventional Ethernet communication to transmit time-sensitive data in autonomous vehicles. The IEEE 802.1 TSN task group manages the time-sensitive data communication. The TSN communication classifies data frames into high and low priority. It allows channel preemption of high-priority data frames. Thus, transmission of time-sensitive data with high-priority can be guaranteed [15].

As mentioned earlier, recent IVN generates a lot of data for autonomous driving services. The data is exchanged outside the vehicle with other elements of the vehicular network. The elements of the vehicular network such as vehicles, road side units (RSU), etc., construct Internet of Vehicles (IoV). For intelligent services for vehicles, IoV uses a large amount of diverse vehicle data. For data transmissions, the IoV allows Vehicle-to-Vehicle (V2V), Vehicle-to-Infrastructure (V2I), Vehicle-to-Pedestrian (V2P) communication, etc. The Vehicle-to-X (V2X) communication uses Cellular-based communication technology (e.g., LTE and 5G) and IEEE 802.11 based dedicated short-range communication (DSRC) technology to communicate each other [16,17,18]. The IoV data is classified into two categories according to the location of occurrence: on-board data and on-road data. On-board data is generated by sensors in vehicles and flooded to vehicular networks, whereas on-road data is generated in road side units; it is the environmental data of roads to represent the current traffic status. The data generated in IoV constitute big data and are used for intelligent services of autonomous vehicles [18,19].

The IoV data flows into the IVN through the vehicle’s network gateway and is delivered to in-vehicle devices required for intelligent services. The IVN’s on-board data is also sent to the IoV network through the network gateway. That is, the vehicular network consists of heterogeneous networks of IoV and IVN connected by the network gateway. In this heterogeneous networks, various large scale data exchanges take place. In addition, incoming and outgoing data traffic in a vehicle are transmitted over an Ethernet backbone in IVN. As mentioned earlier, even if the IVN backbone provides enough bandwidth to process large amounts of data, it has limitations on data explosively flowing into the backbone. Therefore, traffic exchange between IoV and IVN is required considering the capacity and efficiency of the IVN backbone. Because the backbone utilization of large volume data transmission by cameras and LiDAR sensors is increasing in vehicles, if the IoV data from outside a vehicle flows into the backbone without taking this into account, the traffic intensity increases and the probability of a traffic collision in transmission increases. In addition, when the vehicle is sending data traffic to the IoV, wireless state of the IoV should be taken into account. If data traffic for the IoV is transmitted in bad wireless conditions, transmission efficiency decreases and other network elements cannot receive the sent data. Therefore, for autonomous driving, network integration of IVN and IoV considering each network condition is very important. To efficiently exchange data traffic between IVN and IoV, data traffic should be delivered when each network condition is not bad.

*Problems:*

▪ *For autonomous driving services, frequent data exchange occurs in IVN and IoV, but the integrated network environment is not considered in the current situation of the vehicular network.*
▪ *Transmission efficiency is degraded if the network conditions of both IVN and IoV are not considered when transmitting data.*

In this paper, we attempt to solve these problems of the vehicular networks by designing a network gateway. We propose a network gateway in a vehicle to control incoming IoV traffic and outgoing IVN traffic depending on the state of the IVN backbone and IoV. Thus, the proposed network gateway can avoid the transmission efficiency degradation caused by busty traffic in the IVN backbone and bad wireless conditions in IoV. Until now, there have been many studies on vehicle gateways, but IoV and IVN-integrated heterogeneous network environments have not been covered. In terms of intelligent autonomous driving services, both IoV and IVN data will be widely used, and for this, automotive networks must be considered as an integrated heterogeneous network rather than an individual network (i.e., IVN or IoV). The proposed method in this paper provides traffic management of the network gateway in the integrated network architecture of IoV and IVN. Thus, the proposed method has an advantage in terms of transmission efficiency in data exchange between IVN and IoV.

The remainder of this paper is organized as follows. In Section 2, background and related work on IVN and IoV architectures is described. In Section 3, the proposed network access gateway in the integrated network is explained. In Section 4, a performance evaluation for the proposed method is carried out by computer simulation. Finally, Section 5 concludes this paper. 

## 2. Background and Related Work

### 2.1. Background

#### 2.1.1. In-Vehicle Network (IVN)

**Figure 1:**
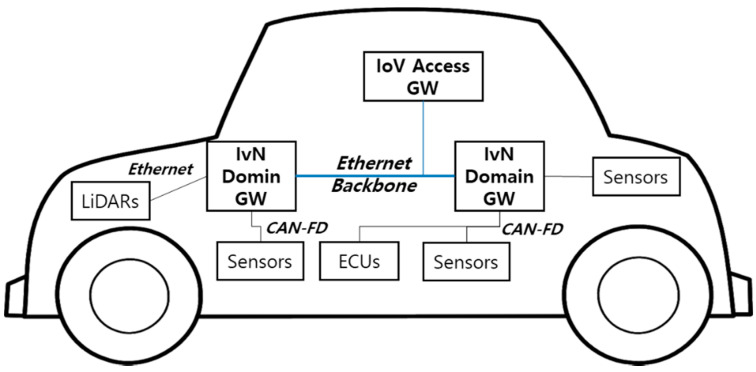
In-vehicle network architecture.

Autonomous vehicles use data generated from cameras and LiDAR sensors to perform machine learning. To obtain more data for improved object recognition, autonomous vehicles attempt to apply high resolution cameras or multi-channel LiDAR sensors. In addition, they attempt to combine several cameras or multi-channel LiDAR sensors [2,3]. Thus, massive amounts of data are generated and transmitted from the vehicle devices. To satisfy the high bandwidth that is required for this type of data transmission, Ethernet is used for the IVN backbone. The Ethernet backbone connects the domain controllers in a vehicle. There are several domain controllers for diagnosis, powertrain, chassis, advanced driver assistance system (ADAS), Infotainment, etc. [4,5,11]. Each domain controller generates data and controls in-vehicle modules. It also acts as a domain gateway. Each domain gateway connects to the IVN backbone and its domain sensors and actuators. Thus, it provides inter-domain communication and intra-domain communication. As mentioned earlier, inter-domain communication uses the Ethernet backbone (e.g., 100Base-T1/1000Base-T1) because of high bandwidth requirements for data transmission. Intra-domain communication can use various in-vehicle communication technologies (e.g., LIN, CAN, CAN-FD, FlexRay, MOST, and Ethernet) according to domain requirements. In case of ADAS and Infotainment domain, Ethernet is mainly used for data transmission as the demand for high-bandwidth data transmission has rapidly increased recently. Figure 1 represents the IVN architecture.

#### 2.1.2. Internet of Vehicles (IoV)

**Figure 2:**
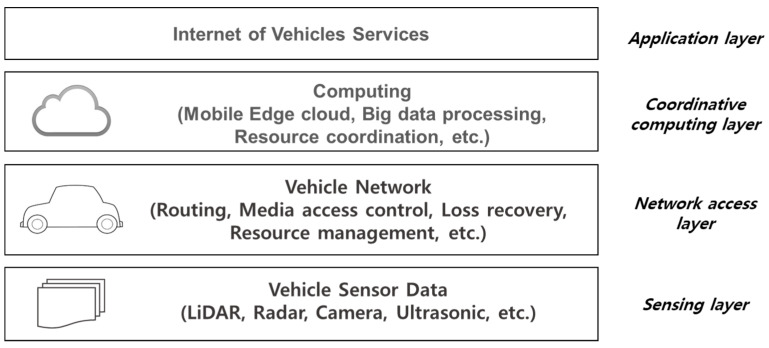
IoV system architecture [20].

In IoV environments, vehicles, road side units, and pedestrians become nodes to join a wireless network. They are connected in a mesh network topology and construct V2X communication networks using various wireless access technologies. As a vehicle connected network, IoV is similar to the Internet of Things (IoT). However, in IoV, the interaction between the nodes (i.e., vehicles) and the users is more important than in IoT. The interaction is reflected in the IoV system architecture. The IoV system architecture is composed of sensing, network access, coordinative computing, and application [20]. In the sensing layer, vehicles sense on-board data from vehicle devices and road side units (RSUs) measure on-road data. The collected data in the IoV nodes is shared through the network access layer. The coordinative computing layer processes the shared data over the network and handles interactions between vehicles and users. Various types of applications are served in the application layer. Figure 2 represents the IoT system architecture.

**Figure 3:**
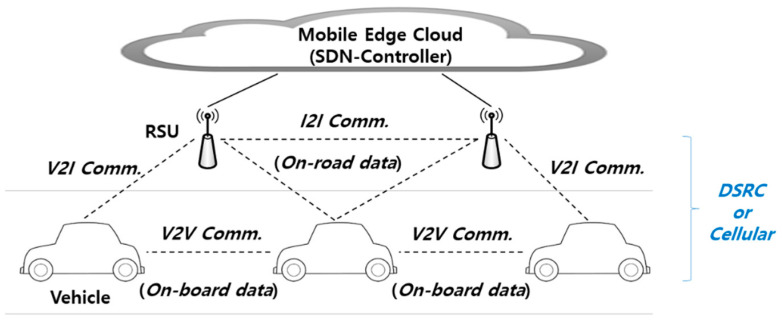
IoV network.

For IoV networks, there are two types of communication technology: Dedicated short range communication (DSRC) and Cellular. DSRC provides high-speed transmission based on IEEE 802.11, but contrary to Cellular, has difficulty guaranteeing stable connectivity. Cellular provides reliable connectivity and high mobility, but this comes at a cost. These two communication technologies are complementary for IoV services. In addition, attempts are being made to combine with edge clouds to support service-based computing of IoV. Edge cloud provides several functionalities (i.e., computing, storage, data processing, etc.) to mobile nodes and supports the coordinative computing layer. In addition, network control efficiency can be improved by building a software-defined network (SDN) through the edge cloud [16,20,21,22,23]. Figure 3 represents the IoV network.

As shown in Figure 3, IoV nodes are connected in various ways and generate on-board data and on-road data for services. This big data includes large scope information in vehicular networks. Through V2X connectivity, the big data flows into the vehicles [18]. This kind of data is used for intelligent transport system (ITS) services. However, as mentioned earlier, in a situation where high-speed transmission of large amounts of data inside the vehicle is expanding, the inflow of large amounts of data from outside the vehicle may reduce the efficiency the in-vehicle backbone. Therefore, it should be considered to manage data traffic in a heterogeneous network environment in which the inside and outside of the vehicle are integrated.

### 2.2. Related Work

A gateway in the vehicular network has two types: IoV gateway and IVN gateway. An IoV gateway is used to connect the IoV network. In [24], a vehicle near edge cloud becomes the IoV gateway and is used as a relay to connect to other vehicles by edge cloud. In [25], the IoV gateway integrates IVN with IoV. It serves to deliver data generated by IVN, which consists of CAN and FlexRay, to IoV. The IVN data is transmitted to edge cloud and is used for self-diagnosis services. An IVN gateway is used for the purpose of connecting domain networks inside a vehicle. As mentioned in previous section, a vehicle has several domains and different domain networks (i.e., CAN, FlexRay, Ethernet, etc.) are used. For safety control of a vehicle, each domain data must be shared and the data is shared through the IVN gateway. Lee et al. provides the gateway to connect FlexRay and Ethernet network and emphasizes the importance of the Ethernet backbone in future vehicle systems [1]. Jeong et al. provides the integrated gateway for four IVN communications: FlexRay, CAN, Ethernet, and MOST [5]. This gateway connects to the onboard-diagnostic (OBD) interface and IVN data is delivered to the interface.

As mentioned earlier, in a current vehicular network, a large amount of data is generated inside and outside the vehicle to support autonomous services. As data traffic explodes in a vehicle, congestion at communication links becomes an important issue and this issue is addressed as a key element in vehicle ad-hoc networks [26]. However, it does not reflect current situation of the vehicular networks. The vehicle ad-hoc networks only considers the outside network of the vehicle. In addition, IVN and IoV gateways are not considering an integrated network environment for IVN-IoV. If the integrated vehicle network is not considered, data transmission efficiency is reduced due to frequent congestion when busty traffic is generated inside/outside the vehicle. This can affect vehicle safety. Therefore, traffic control for IVN communication in the integrated vehicle network must be considered.

## 3. IoV Access Gateway in the Integrated Heterogeneous Network for Vehicles

### 3.1. IoV Access Gateway Design

**Figure 4:**
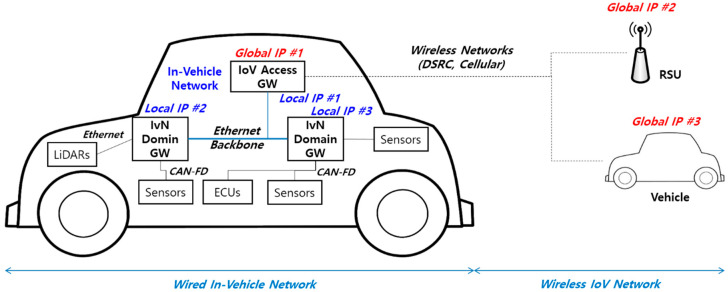
Integrated heterogeneous network for vehicles.

The IoV access gateway (A-GW) is a network gateway that connects the in-vehicle network and the out-of-vehicle network. On-board data generated from in-vehicle devices passes through the IoV access gateway to connect with the IoV network. The IoV access gateway is also used for vehicles to receive on-board data from other vehicles or on-road data from RSUs. For IoV services, a heterogeneous network consisting of IVN and IoV is created through the IoV access gateway. Figure 4 represents the integrated heterogeneous network for IoV services.

As mentioned earlier, the IoV A-GW connects to domain gateways using the Ethernet backbone (IEEE 100Base-T1/1000Base-T1). For IoV network access, it has a global IP address and exchanges on-board and on-road data with other vehicles or RSUs. Inside the vehicle, a local IP network can be constructed over the Ethernet backbone. The address of a domain gateway is mapped to the IoV A-GW. Incoming IoV data with IP can easily arrive at the domain gateway via the IoV A-GW. Outgoing IVN data can be also easily sent to the outside of the vehicle via the IoV A-GW. Even if the infra-domain network does not use IP, the domain gateway provides an Ethernet based IP overlay network, whereby the IVN on-board data exchange to the IoV network can be easily performed. In this heterogeneous vehicular network, the separation of IoV and IVN through the IoV A-GW also has security advantages. Without connecting external data directly to the domain gateway in the IVN backbone, IoV A-GW can check for intrusion detection more efficiently.

The IoV A-GW has two types of interfaces for IVN and IoV. Through the interfaces, large amounts of data between the wired in-vehicle network and the wireless IoV network are exchanged. In the IVN, on-board data is used to control autonomous driving or to share its vehicle information with other vehicles. If the traffic intensity of the Ethernet backbone is increased, information transmission for autonomous driving control is delayed, and thus the vehicle safety is seriously affected. Therefore, it is necessary to manage the transport traffic in consideration of the state of the IVN backbone. In particular, external data is required for various IoV services, but the direct relationship with vehicle driving control and safety is less than in-vehicle data from vehicle sensors. Thus, IoV A-GW should process in-vehicle data and IoV data separately, and it should be able to control incoming traffic (i.e., into the vehicle) from IoV networks.

**Figure 5:**
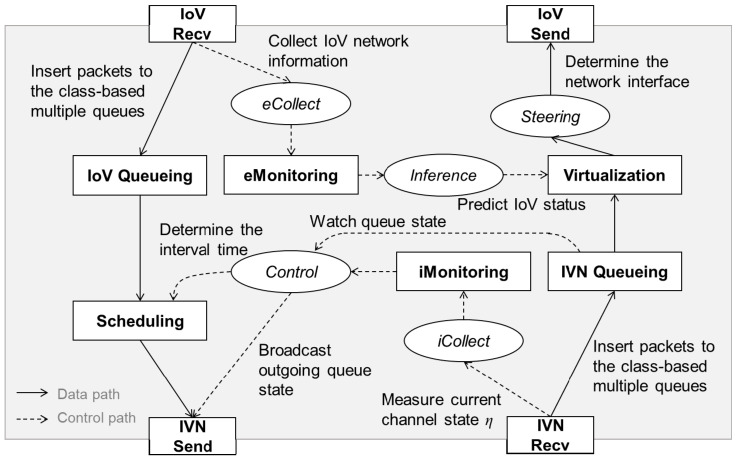
The proposed IoV access gateway (A-GW) system architecture.

Figure 5 represents the proposed IoV A-GW system architecture. There are four states for the network interfaces (i.e., *IoV Recv*; *IVN Send*; *IoV Send*; *IVN Recv*), three states for the incoming IoV flow (i.e., *IoV Queueing*; *eMonitoring*; *Scheduling*) and three states for the outgoing IVN flow (i.e., *IVN Queueing*; *iMonitoring*; *Virtualization*). The result of *eMonitoring* state is used for the outgoing flows and the result of *iMonitoring* state is used for the incoming flows. Five functions support state operations. *eCollect* and *iCollect* functions collect data from the incoming and outgoing data flows. *Control* and *Steering* functions adjust traffic flows. *Inference* function provides predicted information of the IoV to the outgoing flows. The proposed A-GW integrates the IVN and the IoV, and controls the traffic flows according to each network condition.

Data traffic received from the *IoV Recv* state flows into the IVN Ethernet backbone through *IVN Send* state via *IoV Queueing* and *Scheduling* states. In the *IoV Queueing* state, incoming data packets are classified by using class-based multiple queues according to priority. In the *Scheduling* state, data packets in multiple queues are scheduled to be sent to the IVN backbone. The scheduling interval for each queue is determined by the *Control* function. The *Control* function uses information on the *iMonitoring* and *IVN Queueing* state. The current backbone state is reported from *iCollect* function. This information is delivered through the control path. The *iCollect* function measures the channel utilization by listening to the channel for given time; that is, it monitors busy channel time to obtain the channel usage rate. If it is difficult to measure the channel utilization time by carrier sensing, the channel state can be measured by the response time of each domain GW for a control packet issued by the IoV A-GW. With the information, the usage rate of the Ethernet backbone in the vehicle can be calculated. Then, it is possible to flow external data from the IoV into the IVN backbone considering the Ethernet backbone efficiency.

On-board data from in-vehicle sensors received by the *IVN Recv* state is sent to *IoV Send* state via *IVN Queueing* and *Virtualization* states. In the *IVN Queueing* state, data packets are queued in multiple class-based queues according to their priority. In the *Virtualization* state, a scheduled data packet is sent using virtualized IoV network. According to wireless IoV network conditions, a network interface can be switched. For traffic steering in the IoV network, the network condition should be monitored and predicted in the wireless condition. Thus, the proposed IoV A-GW collects the external network information using the *eCollect* function. In the *eMonitoring* state, the wireless condition of the external IoV network is monitored. The *Inference* function predicts the IoV wireless conditions by performing machine learning such as the deep neural network algorithm. Then, according to the external network status, data traffic generated by in-vehicle sensors and flowing into the IoV network can be steered. The IoV network is virtualized, and data packets are delivered to the IoV through the virtualized network. The *Steering* function controls IoV network interfaces through the inference results. Then, data traffic can be transmitted without consideration of IoV network selection. That is, data traffic is delivered to virtualized IoV network and IoV network selection is performed by the *Steering* function.

### 3.2. Traffic Control for the IVN Ethernet Backbone

The IVN backbone has several types of data flows: internal-to-internal, internal-to-external, and external-to-internal. The internal-to-internal flow is mainly the flow of vehicle control data, and is the most important flow. The external-to-internal flow refers to the transmission of on-board and on-road data flowing from the IoV network outside the vehicle. In addition, the internal-to-external flow means that on-board data inside the vehicle is transmitted to the IoV network outside the vehicle. As mentioned above, vehicles use the IVN backbone to receive external on-board/on-road data and to transmit internal on-board data. The resource of the IVN backbone is limited, and the traffic in the vehicle network is increasing. Therefore, managing the traffic flows considering the IVN backbone status should be provided. Transmission of the highest priority internal-to-internal flow must be guaranteed, and the other flows can be controlled according to the IVN backbone status.

For the external-to-internal flow, the packet scheduling interval is controlled, as shown in Algorithm 1. The channel utilization (*η*) is compared with two threshold values (*Z_b_*, *Z_u_*) to determine the scheduling interval. If the channel utilization is greater than the lower threshold (*Z_b_*), a scheduling delay occurs by selecting a random number *n* and multiplying the slot time *TS*. The high channel utilization in the IVN backbone means that a lot of in-vehicle data is transmitted. In-vehicle data is usually high-priority data related to vehicle control. Thus, it should not be disturbed in transmission by the incoming traffic. Through the scheduling delay, collision between the incoming data and the in-vehicle data can be avoided. If the channel utilization is greater than the upper threshold (*Z_u_*), the scheduling is stopped for a while. The upper threshold means that the transmission of incoming traffic is impossible due to a very high channel utilization. In this case, the transmission of incoming data to the IVN backbone is stopped until the channel utilization is lowered.
**Algorithm 1.** Traffic control at IoV A-GW.*TS**Traffic*−*Control(η, Z_b,_ Z_u_)**1*: *d ← base_time**2*: if *η ≥ Z_b_*:*3*:  *n ← random(1,k)**4*:  *d ← d + TS × n**5*: else if *η ≥ Z_u_*: *6*:  *d ← hold**7:* end-if

In the internal-to-external flow, when the use of the backbone channel is high due to IVN internal data transmission, domain gateways can reduce backbone congestion by delaying the transmission of outflow data to the IoV network. Each domain gateway measures the channel utilization in the same manner as the IoV A-GW, and adjusts transmission traffic by applying such channel access delays as those shown in Algorithm 1. If the domain gateways cannot measure the channel state, the channel state information can be applied by receiving the channel state value broadcasted by IoV A-GW.

### 3.3. Traffic Steering for IoV Network

For IoV networks, several wireless technologies are used. Through network virtualization, a wireless network with optimum conditions can be selected and used for data transmission. The network virtualization can increase network efficiency for IoV networks by automatically controlling the traffic flow. By integrating with the mobile edge cloud, it is possible to provide wireless network virtualization while reducing the computing loads on vehicles. Although not the scope of this paper, there are several studies on network virtualization for IoV networks [27,28,29].

**Figure 6:**
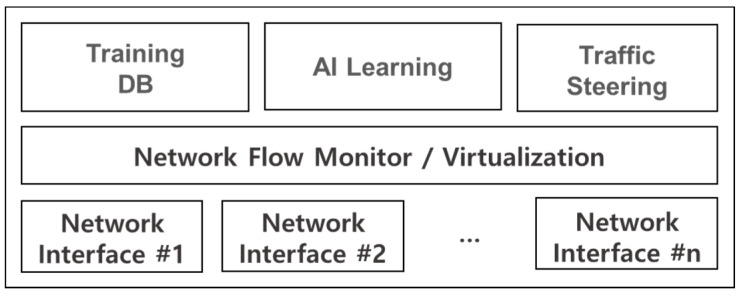
Traffic control architecture using machine learning [33].

In addition, the optimal state of a wireless network can be known through machine learning. In general, wireless network state changes frequently and is highly affected by noise or interference. Thus, it is necessary to be aware of the network condition and then attempt to transmit data. To do this, network condition inference using machine learning is performed. The IoV A-GW collects information from IoV networks in the *eMonitoring* state shown in Figure 5. The information is used as training data to infer such network conditions as link quality or congestion. Several algorithms can be used for the training: logistic regression, Bayesian classifier, SVM, deep neural network, etc. As a result of machine learning, the state of wireless conditions of IoV can be predicted [30,31,32,33]. In the proposed method, the deep neural network model for network status prediction of our previous work [33] can be applied. The deep neural network model is composed of 1 input with 2 nodes, 8 hidden layers with 30 nodes and 1 output with 1 node [33]. As the input data, network throughput and received signal strength can be used. If the wireless network is selected according to the predicted value in the *Virtualization* state, the IoV transmission efficiency can be increased. The traffic control architecture using machine learning is as shown in Figure 6.

## 4. Performance Evaluation

For performance evaluation, computer simulations are used. The simulator is implemented by C-language using SMPL library [34]. The SMPL library provides event-driven environments during computer simulation. The proposed IoV access gateway is compared to a conventional IoV access gateway through the event-driven computer simulation. The proposed method recognizes the IVN backbone state and controls incoming traffic into the IVN backbone as shown in Figure 5. However, the conventional method does not consider the IVN backbone state. The computer simulation is performed under the simulation environments in Section 4.1 and the IVN backbone link model in Section 4.2.

### 4.1. Evaluation Environments

There are three kinds of data traffic in the simulation: IVN background traffic, traffic provided by IoV, and traffic serving into IoV. IVN background traffic has highest priority for driving vehicles. Traffic provided by IoV and traffic serving into IoV have two types of priority: high and low. Their high-priority is lower than that of IVN background traffic. Each traffic generation occurs in an exponential distribution with 100 msec as a mean time. In the traffic for IoV (i.e., incoming traffic from IoV and outgoing traffic into IoV), it is assumed that high-priority traffic of 30%, 50%, and 70% is generated. Packets are scheduled to send to the IVN backbone, and a weighted fair queueing (WFQ) scheduling mechanism is used. The weight ratio of the scheduler is set to three for IVN background packets, one for incoming packets, and one for outgoing packets. When the traffic is generated, the packet size is 1500 Bytes and a single traffic flow is composed with 10 packets. The IVN backbone link is assumed to be a 100 Mbps Ethernet link. The link error is set to 1%. If the link error causes packet loss, retransmission is attempted twice through the binary random backoff mechanism. For the proposed method, *Z_b_* and *Z_u_* are set to 0.6 and 0.9, respectively. As mentioned earlier, the proposed IoV A-GW exploits the traffic control in Algorithm 1. In the proposed method, *k* is set to 100 and *TS* becomes 12 msec for a single packet transmission. In addition, *hold* is set to 100, which is the same value as *k*. The simulation is performed for 1 h. Table 1 represents simulation parameters.

### 4.2. IVN Backbone Link Model

**Figure 7:**
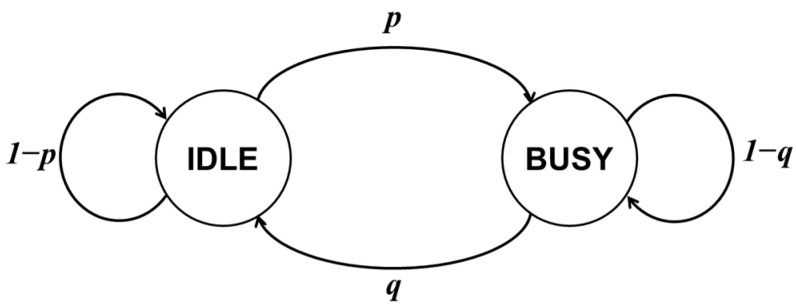
IVN Ethernet backbone link model.

The IVN Ethernet backbone is modeled by two-state Markov chain [35] with an idle and a busy state, as shown in Figure 7. In the busy state, because the backbone channel is used, data cannot be transmitted. In the idle state, the channel is not used, and thus data transmission is attempted after carrier sensing. At this time, the probability of packet loss due to collision is assumed to be 1%. The Markov chain is a probabilistic model in which state transition occurs according to given probability. The transition probability *p* from the idle state to the busy state is set to 0.4, 0.5, and 0.6. The transition probability *q* from the busy state to the idle state is set to 0.7. In addition, it is assumed that the state transition of the backbone channel occurs every 5 msec.

### 4.3. Simulation Results

As mentioned earlier, the proposed method considers the IVN backbone states. Thus, if the probability of collision during transmission increases due to frequent use of the backbone channel, channel access to the backbone channel is avoided. The conventional method does not consider the backbone channel state—it just sends data packets if the channel is idle after carrier sensing. To evaluate the performance of the proposed method, transmission delays for IoV network traffic (i.e., both incoming from IoV and outgoing to IoV) are measured. The transmission of IVN traffic is guaranteed, and it is assumed that it is transmitted with the highest priority without collision. The IVN traffic comprises ECUs and sensors data transmitted between domain gateways in a vehicle. Therefore, the transmission must be guaranteed because it is related to driving safety. However, IVN traffic can allow delays and loss. The transmission delay refers to the time until the scheduled data is successfully transmitted. If collision in the IVN backbone occurs during data transmission, binary random backoff, and retransmissions are performed, and the transmission delay due to retransmission increases.

**Figure 8:**
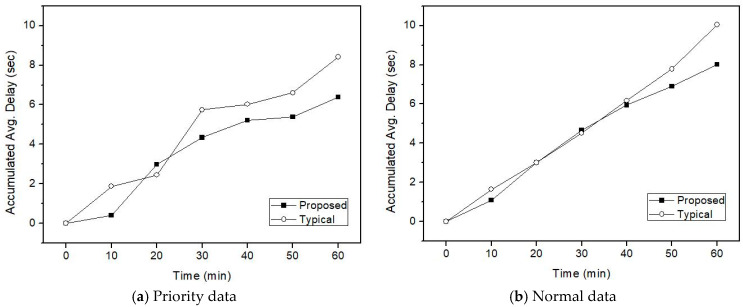
Transmission delay when *p* is 0.4 and IoV high-priority is 30%.

**Figure 9:**
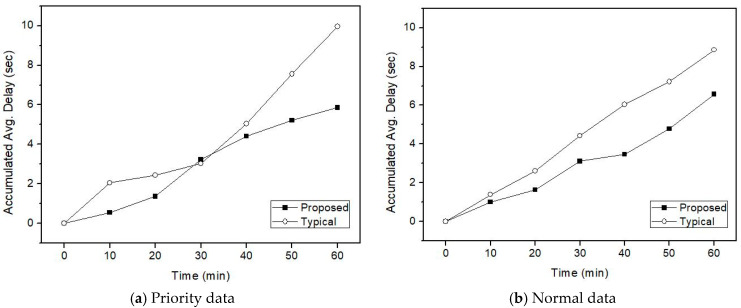
Transmission delay when *p* is 0.5 and IoV high-priority is 30%.

**Figure 10:**
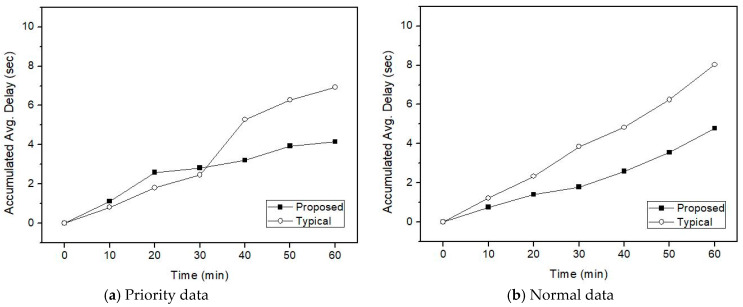
Transmission delay when *p* is 0.6 and IoV high-priority is 30%.

Figure 8, Figure 9 and Figure 10 represent transmission delays of priority data and normal data according to the channel transition probability from the idle state to the busy state for the simulation time.

In the proposed IVN A-GW, the transmission delay in both the normal and priority traffic appears smaller than the existing A-GW. The difference in transmission delay increases from 30 min later. By looking at the channel state of the IVN backbone link and avoiding channel congestion as a random backoff algorithm, it is possible to reduce the overall traffic transmission delay. That is, the retransmission delay due to collision during data transmission appeared larger than the delay due to the transmission control according to the channel state. Because the amount of data traffic generated in the heterogeneous vehicular network of IVN and IoV is very large, the difference in transmission delay may be increased. This can affect intelligent vehicle services. Thus, in terms of the proposed IVN A-GW, it is becoming increasingly important to control IoV data traffic while considering the IVN backbone utilization.

**Figure 11:**
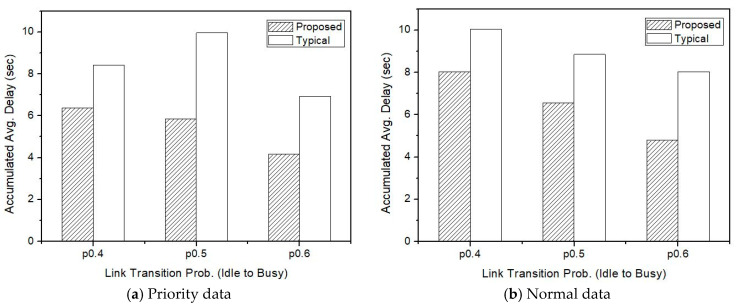
Transmission delay when IoV high-priority is 30%.

Figure 11 shows the overall simulation results according to the transition probability *p*. As shown in Figure 8, Figure 9 and Figure 10, there is a difference in transmission delay of 2 s or more between the proposed method and the existing method. As the link transition probability from the idle state to the busy state increases, the overall transmission delay is slightly reduced. It is shown that the actual transmission amount has decreased due to the decrease in the idle state for data transmission, and thus the total accumulated transmission delay for the simulation time is reduced.

**Figure 12:**
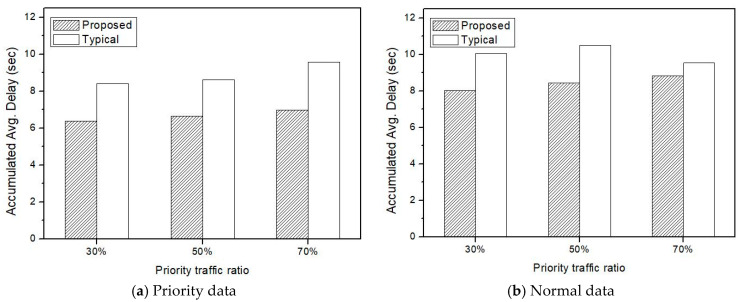
Transmission delay according to IoV high-priority ratio.

Figure 12 shows the overall simulation results according to IoV high-priority ratio. Even if priority traffic increases, transmission delay in the proposed method is reduced in both high-priority and normal traffic. In particular, the proposed method shows better performance in processing high-priority traffic than normal traffic when the proportion of high-priority traffic in a vehicle network is large.

## 5. Conclusions

A vehicular network for autonomous driving services consists of IVN and IoV. In the former, massive data is generated from cameras, LiDAR sensors, and infotainment multimedia. Thus, a high-speed backbone is required, therefore, an Ethernet backbone is used for this kind of data transmission. Moreover, in IoV, a large number of nodes (i.e., vehicles, RSUs, etc.) comprise a network and exchange various types of data. Thus, the IoV data is fed into or out of the vehicle. Even though the IVN backbone uses a high-speed communication network, in a situation where the utilization is high due to the data generated in the IVN, effective processing of IoV data in the IVN is required. That is, in a vehicle, IVN local data transmission for driving safety should be guaranteed, and IoV data transmission should be provided in consideration of the utilization state of the IVN backbone. The proposed method, the backbone channel access time for data transmission is adjusted according to the utilization state of the IVN backbone. As a result, the time spent on IoV data transmission in the IVN backbone was reduced. The IVN backbone efficiency is improved by reducing the transmission delay for both IoV normal data and priority data.

## Figures and Tables

**Table 1:** Simulation parameters.

Parameters	Values
Traffic generation	Exponential dist. (100 msec)
IoV high-priority	30%, 50%, 70%
Scheduling	WFQ
Scheduling weight ratio	3:01:01
Packet size	1500 Bytes
# of packets per a single flow	10
IVN backbone	Ethernet (100 Mbps)
Link error	1%
Retry counts	2
*Z_b_*: *Z_u_*	0.6:0.9
*k*, *hold*	100
Transition prob. from idle to busy (*p*)	0.4, 0.5, 0.6
Transition prob. from busy to idle (*q*)	0.7
Simulation time	1 h

## Data Availability

Data available on request due to restrictions.

## Abbreviations

The following abbreviations are used in this manuscript.

**Abbreviation**	**Description**
IVN	In-vehicle network
IoV	Internet of Vehicles
ECU	Electric control unit
LiDAR	Light detection and ranging
LIN	Local interconnect network
CAN	Controller area network
CAN-FD	CAN—flexible data rate
MOST	Media-oriented system transport
CSMA/CD	Carrier sense multiple access/collision detection
TSN	Time = sensitive networking
V2X	Vehicle-to-x
V2V	Vehicle-to-Vehicle
V2I	Vehicle-to-Infrastructure
V2P	Vehicle-to-Pedestrian
I2I	Infrastructure-to-Infrastructure
DSRC	Dedicated short-range communication
IoT	Internet of Things
RSU	Road side unit
ITS	Intelligent transport system
A-GW	Access gateway
SDN	Software defined network

## Notations

The following notations are used for Algorithm 1.

**Notation**	**Description**
*d*	Scheduling delay
*η*	Channel utilization
*Z_b_*	Lower threshold
*Z_u_*	Upper threshold
*TS*	Slot time

## References

[1] Lee T.-Y., Lin I.-A., Liao R.-H. (2020). Design of a FlexRay/Ethernet Gateway and Security Mechanism for In-Vehicle Networks. Sensors.

[2] Guan L., Chen Y., Wang G., Lei X. (2020). Real-Time Vehicle Detection Framework Based on the Fusion of LiDAR and Camera. Electronics.

[3] Jung J., Park M., Cho K., Mun C., Ahn J. (2020). Intelligent Hybrid Fusion Algorithm with Vision Patterns for Generation of Precise Digital Road Maps in Self-driving Vehicles. Ksii Trans. Internet Inf. Syst..

[4] Wu Z., Zhao J., Zhu Y., Lu K., Shi F. (2019). Research on In-Vehicle Key Management System under Upcoming Vehicle Network Architecture. Electronics.

[5] Jeong Y., Son S., Jeong E.H., Lee B.K. (2018). A Design of a Lightweight In-Vehicle Edge Gateway for the Self-Diagnosis of an Autonomous Vehicle. Appl. Sci..

[6] Xiao Z., Li Y. Hardware Design of Automobile Door with Local Interconnect Network Bus. In Proceedings of International Conference on Control, Automation and Systems Engineering (CASE).

[7] Lee S.Y., Park S.H., Choi H.S. (2013). Implementation of Automotive Media Streaming Service Adapted to Vehicular Environment. Lect. Notes Electr. Eng. (Lnee).

[8] Hagiescu A., Bordoloi U.D., Chakraborty S., Sampath P., Ganesan P.V.V., Ramesh S. Performance Analysis of FlexRay-based ECU Networks. In Proceedings of ACM/IEEE Design Automation Conference.

[9] Law D., Dove D., D’Ambrosia J., Hajduczenia M., Laubach M., Carlson S. (2013). Evolution of Ethernet Standards in the IEEE 802.3 Working Group. IEEE Commun. Mag..

[10] Lee J., Park S. (2019). Time-Sensitive Network (TSN) Experiment in Sensor-Based Integrated Environment for Autonomous Driving. Sensors.

[11] Wu W., Li R., Xie G., An J., Bai Y., Zhou J., Li K. (2020). A Survey of Intrusion Detection for In-Vehicle Networks. IEEE Trans. Intell. Transp. Syst..

[12] Son S., Jeong Y., Lee B.K. (2019). An Optimal Driving Support Strategy (ODSS) for Autonomous Vehicles based on an Genetic Algorithm. Ksii Trans. Internet Inf. Syst..

[13] IEEE 802.3bw (100BASE-T1) Task Force. https://www.ieee802.org/3/bw/.

[14] IEEE 802.3bp (1000BASE-T1) Task Force. https://www.ieee802.org/3/bp/.

[15] IEEE 802.1 TSN Task Group. https://1.ieee802.org/tsn/.

[16] Storck C.R., Duarte-Figueiredo F. (2019). A 5G V2X Ecosystem Providing Internet of Vehicles. Sensors.

[17] Kim D.Y., Kim S. (2019). A Data Download Method from RSUs using Fog Computing in Connected Vehicles. Comput. Mater. Contin..

[18] Xu W., Zhou H., Cheng N., Lyu F., Shi W., Chen J., Shen X. (2018). Internet of Vehicles in Big Data Era. IEEE/CAA J. Autom. Sin..

[19] Gwak J., Jung J., Oh R., Park M., Rakhimov M.A.K., Ahn J. (2019). A Review of Intelligent Self-Driving Vehicle Software Research. Ksii Trans. Internet Inf. Syst..

[20] Yang F., Li J., Lei T., Wang S. (2017). Architecture and Key Technologies for Internet of Vehicles: A Survey. J. Commun. Inf. Netw..

[21] Shrestha R., Bajracharya R., Nam S.Y. (2018). Challenges of Future VANET and Cloud-based Approaches. Wirel. Commun. Mob. Comput..

[22] Roman R., Lopez J., Mambo M. (2018). Mobile Edge Computing, Fog et al.: A Survey and Analysis of Security Threats and Challenges. Future Gener. Comput. Syst..

[23] Li Z., Zhu Q. (2020). An Offloading Strategy for Multi-User Energy Consumption Optimization in Multi-MEC Scene. Ksii Trans. Internet Inf. Syst..

[24] Abbas M.T., Muhammad A., Song W.C. (2020). SD-IoV: SDN enabled Routing for Internet of Vehicles in Road-aware Approach. J. Ambient Intell. Humaniz. Comput..

[25] Jeong Y.N., Son S.R., Lee B.K. (2019). The Lightweight Autonomous Vehicle Self-Diagnosis (LAVS) Using Machine Learning Based on Sensors and Multi-Protocol IoT Gateway. Sensors.

[26] Khan U.A., Lee S.S. (2019). Multi-Layer Problems and Solutions in VANETs: A Review. Electronics.

[27] Kim D.Y., Park J.H., Lee Y., Kim S. (2020). Network Virtualization for Real-Time Processing of Object Detection using Deep Learning. Multimed. Tools Appl..

[28] Moubayed A., Shami A. (2020). Softwarization, Virtualization, & Machine Learning For Intelligent & Effective V2X Communications. IEEE Intell. Transp. Syst. Mag..

[29] Tayyaba S.K., Khattak H., Almogren A., Shah M.A., Din I.U., Alkhalifa I., Guizani M. (2020). 5G Vehicular Network Resource Management for Improving Radio Access Through Machine Learning. IEEE Access.

[30] Kim S., Kim D.Y., Park J.H. (2018). Traffic Management in the Mobile Edge Cloud to Improve the Quality of Experience of Mobile Video. Comput. Commun..

[31] Zhang C., Patras P., Haddadi H. (2019). Deep Learning in Mobile and Wireless Networking: A Survey. IEEE Commun. Surv. Tutor..

[32] Nguyen T.T.L., Pham T.M. Optimization Model and Algorithm for Dynamic Service Aware Traffic Steering in Network Functions Virtualization. In Proceedings of IEEE International Conference on Communications and Electronics.

[33] Kim D.Y., Kim S. (2020). Network-Aided Intelligent Traffic Steering in 5G Mobile Networks. Comput. Mater. Contin..

[34] MacDougall M.H. (1987). Simulating Computer Systems, Techniques and Tool.

[35] Trivedi K.S. (2002). Probability and Statistics with Reliability, Queuing and Computer Science Application.

